# Relative energy deficiency in sport: a cross-sectional study of nutritional, biochemical and hormonal profiles in Czech female endurance athletes at risk of low energy availability

**DOI:** 10.3389/fspor.2026.1663582

**Published:** 2026-02-05

**Authors:** Jana Woronyczová, Miroslava Nováková, David Gerych, Kateřina Jurková, Čestmír Oberman, Emil Bolek, Jaroslav Pilný, Libor Vítek

**Affiliations:** 1 Institute of Medical Biochemistry and Laboratory Diagnostics, 1st Faculty of Medicine, Charles University and General University Hospital in Prague, Prague, Czechia; 2 Sports Research Institute of the Czech Armed Forces, Prague, Czechia; 3 Faculty of Physical Education and Sport, 1st Faculty of Medicine, Charles University, Prague, Czechia; 4 Institute of Anatomy, Faculty of Medicine in Hradec Kralove, Charles University, Prague, Czechia; 5 4th Department of Internal Medicine, 1st Faculty of Medicine, Charles University and General University Hospital in Prague, Prague, Czechia

**Keywords:** female athletes, LEA, LEAF-Q, low energy availability, metabolic and hormonal markers, RED-S syndrome

## Abstract

**Introduction:**

Regular physical activity is associated with substantial health benefits, provided the body has sufficient energy sources. However, a long-term low-calorie intake can cause the syndrome of Relative Energy Deficiency in Sport (RED-S) with a significant health threat to athletes. Therefore, the objective of the current study was to evaluate markers of RED-S in a cohort of female athletes.

**Methods:**

The study was carried out in a cohort of female endurance athletes (*n* = 23) and healthy female control subjects (*n* = 21) recruited from a total of 42 athletes and 45 controls who underwent the Low Energy Availability in Females Questionnaire (LEAF-Q) survey. Anthropometric, nutritional, and laboratory analyses were performed on study subjects.

**Results:**

A higher LEAF-Q score signifying low energy availability (LEA) was observed in athletes compared to controls (8 vs. 5, *p* < 0.005). Menstrual problems were significantly more common in athletes, with 33.3% of athletes having amenorrhea (*p* < 0.001). Compared to control women with LEAF-Q < 8, energy expenditure was higher (*p* < 0.001) in athletes with LEAF-Q ≥ 8, and athletes had a negative energy balance (90%) with a very low value of energy availability (24.3 kcal/kg FFM/day). Athletes with a LEAF-Q score ≥8 had lower serum concentrations of estradiol (*p* < 0.001), progesterone (*p* < 0.001), leptin (*p* < 0.001), white blood cells (*p* < 0.005) and phosphorus (*p* < 0.005). Furthermore, they had significantly higher concentrations of hepcidin (*p* < 0.05) and free T3 (*p* < 0.05).

**Discussion:**

LEA is prevalent in female endurance athletes, and its diagnosis deserves a multifactorial approach with anthropometric and nutritional analyses, and the use of a wider range of laboratory markers.

## 1 Introduction

The syndrome of Relative Energy Deficiency in Sport (RED-S) refers to impaired physiological functions including, but not limited to, metabolic rate, menstrual function, musculoskeletal health, immunity, protein synthesis, cardiovascular health, affecting both sexes (1). The cause of the syndrome is due to the imbalance between the dietary energy intake and the energy expenditure required to meet the energy demands of the basal metabolism and daily activities, resulting in low energy availability (LEA) (1). LEA is a powerful disruptor and stressor that triggers marked hormonal and metabolic responses. As a result, LEA affects bone formation and reduces synthesis of skeletal muscle proteins. Different tissues and/or systems are affected in many ways, and this response varies between men and women (2). Long-term pathophysiological sequelae of LEA can be enormous, with a profound impact on the human body, health, and physical performance (2). LEA in athletes occurs as a result of intentional changes in body mass, appetite changes, time constraints, or disordered eating behavior and can exist in the presence or absence of eating disorders. High training volumes with reduced opportunities to eat or misguided weight loss practices can contribute to LEA in athletes. There are currently two LEA forms: adaptable and problematic. Adaptable LEA (e.g., scheduled period of intensive training or competition) is associated with benign effects and is typically a short-term experience with minimal (or no) long-term impact on health, well-being, or performance. Problematic LEA is associated with substantial and potentially chronic health and performance impairments (1).

Energy availability (EA) is usually calculated from energy intake and energy expenditure, relative to fat-free mass (FFM). Although EA estimates are used to support a diagnosis of RED-S, there are many problems associated with the measurement and interpretation of EA (3). Laboratory and clinical quantification of EA is difficult and challenging due to significant under- or overestimation of energy intake and/or exercise energy expenditure. Melin et al. proposed values of EA for men and women according to its physiological and clinical impact, from optimal EA >40 kcal/kg FFM/day in men and >45 kcal/kg FFM/day in women, to subclinical LEA (EA = 30–40 kcal/kg FFM/day) and clinical LEA (EA <30 kcal/kg FFM/day) (4).

Questionnaires have been used routinely as a convenient and simple tool to detect and identify risk factors for RED-S. If they indicate any risk of RED-S, clinical follow-up is necessary to prevent progression of the condition and protect the health and performance of athletes. As generally acknowledged, the Low Energy Availability in Females Questionnaire (LEAF-Q) and the Eating Disorder Examination Questionnaire (EDE-Q) are the most widely used validated questionnaires (5). LEAF-Q determines the risk of LEA with high sensitivity but only in women, while EDE-Q serves as a surrogate marker of the risk of LEA in both sexes. EDE-Q appears to be the most widely used psychometric self-report measure for the screening of eating disorders (6), but this method underestimates body weight.

The health risks associated with LEA include nutritional deficiencies, chronic fatigue, an increased risk of infectious diseases, altered functions of the cardiovascular, gastrointestinal, skeletal, renal, or nervous systems (1). Importantly, LEA is known to affect the reproductive system and other interrelated hormonal pathways, resulting in menstrual dysfunction in female athletes. Specifically, LEA reduces the hypothalamic pulsatile release of gonadotropin-releasing hormones, causing functional hypothalamic amenorrhea and leading to decreased bone mass (7). Although various anthropometric, nutritional, laboratory and other diagnostic markers have been proposed as surrogate markers of LEA and RED-S, the biomarkers used in published studies vary and no standardized diagnostic protocol has been generally accepted to identify subjects at risk of LEA so far (1, 8).

Therefore, the objective of the present cross-sectional study was to compare the routinely available nutritional, biochemical, and hormonal variables between endurance athletes at risk of LEA and lightly physically active control participants without LEA to find the most predictive markers.

## 2 Methods

### 2.1 Study subjects

A total of 42 female endurance athletes (23 medium- and long-distance runners and 19 cross-country skiers) and 45 non-athlete light physically active female university students between the ages of 15–30 consecutively examined in the Sports Research Institute of the Czech Armed Forces between 2021 and 2022 were included in the study. Athletes and control women who used hormonal contraceptives, pregnant or lactating women were excluded from the study. Based on the evaluation of the LEAF-Q data and the interest of the subjects examined in the further participation in the study, the individuals were divided into two study groups: endurance athletes at risk of LEA (LEAF-Q score ≥8) and control participants not at risk of LEA (LEAF-Q score <8).

All athletes with LEAF-Q ≥ 8 were interested in additional participation in the study, therefore, the athlete study group with LEAF-Q score ≥8 consisted of 23 women athletes (16–29 years, Table 1), 16 runners, and 7 cross-country skiers. The subjects were representatives of endurance sport disciplines mainly from youth sports centers and all were of Caucasian origin.

**Table 1:** Characteristics of the study groups.

Characteristics	Female athletes with a LEAF-Q score ≥8 (*n* = 23)	Control group with a LEAF-Q score <8 (*n* = 21)	*P*-value
Age (years)	20.3 ± 4.1	22.9 ± 2.3	**<0.05**
Height (cm)	170 ± 5.3	166 ± 5.6	**<0.05**
Weight (kg)	56.8 ± 7	61.5 ± 6.6	**<0.05**
BMI (kg/m^2^)	19.7 ± 2	22.2 ± 1.9	**<0.001**
Body fat (%)	19.9 (12.7–21.7)	22.4 (20.7–25.3)	**<0.005**
Muscle mass (kg)	44.2 ± 4	45.1 ± 4	NS
FFM (kg)	46.6 ± 4.2	47.5 ± 4.2	NS
Training (h/week)	12 (10–15)	5 (4.5–6)	**<0.001**

Data presented as mean ± SD for normal distributed data and as median and interquartile range for skewed data of training frequency. Bold values indicate statistically significant results.

BMI, body mass index; FFM, fat-free mass.

Not all women in the control group who had LEAF-Q < 8 were interested in further participation in the study (14 controls with LEAF-Q < 8 and 10 controls with LEAF-Q ≥ 8 were therefore excluded from further examinations). Therefore, the controls consisted of 21 healthy women with a LEAF-Q score <8 of Caucasian origin (20–30 years, Table 1). The women were lightly physically active (1–3 days a week), and none of the control women had a sports background. These two groups, selected according to the LEAF-Q score, were further examined in detail.

The study was designed to intentionally compare endurance athletes at risk of LEA with lightly physically active control participants without LEA. This approach was chosen to minimize the heterogeneity related to training load and to allow comparison with a physiologically representative reference group.

The whole study was carried out in accordance with the Declaration of Helsinki of 1975, as revised in 1983. The athlete study was approved by the CASRI Ethics Committee (No. 6/1–6/8 2019). All participants signed an informed consent. Before recruitment, all participants received detailed information about study objectives, which was also included in the informed consent form. Participants under 18 years of age were included in this study with parental consent.

### 2.2 Questionnaire survey

LEAF-Q in study participants was used as previously described (9). This standardized questionnaire aims to address symptoms of insufficient energy intake and is used for screening female athletes to identify individuals at risk of the female athlete triad. The cut-off value of LEAF-Q of 8 discriminating those at risk of the female athlete triad was also used in our study (9). The questions of LEAF-Q are divided into three areas that cover injury, gastrointestinal, and reproductive functions. The questionnaire was translated into Czech, uploaded to an online survey platform (Microsoft Forms, Microsoft Corporation, USA), and evaluated and scored as described (9). The LEAF-Q data from the online questionnaire were collected from all participants as described above.

### 2.3 Body composition analysis and energy expenditure

Body composition (weight, BMI, body fat, muscle mass, and FFM) was evaluated using bioelectrical impedance analysis technology on an automatic body composition analyzer (Tanita MC-780, Tanita Corp., Japan). All measurements were made by trained personnel using a standardized technique. The resting metabolic rate (RMR) was not measured directly but was estimated using the Katch & McArdle formula (10). The total daily energy expenditure was also estimated by multiplying RMR by a standard activity factor. We used a uniform factor for athletes of 1.725 (hard exercise/sports 6–7 days a week) and non-athletes of 1.375 (light exercise/sports 1–3 days a week) (11). The selected activity factors were justified based on the characteristics of the training program, including intensity, type, and frequency of exercise. The factor for non-athletes reflect light to moderate physical activity levels typical of recreationally active individuals without a sporting background. Exercise energy expenditure was calculated as the energy expenditure per day minus RMR. EA reflects the difference in energy intake and exercise energy expenditure in relation to FFM.

### 2.4 Dietary analysis

Participants enrolled according to LEAF-Q scores completed dietary records using the NutriData.cz web application (Fitsport-komplex s.r.o, Czech Republic). Dietary intake was recorded for a minimum of four complete days, including at least one weekend day, within a 14-day period in order to capture habitual intake and potential variability. Participants were instructed to record all foods and beverages consumed, including portion sizes. Portion sizes were estimated using household measures or standardized portion size guides provided within the application. Dietary records were subsequently analyzed using NutriPro nutritional software (Fitsport-komplex s.r.o, Czech Republic). Total energy intake and macronutrient intake were calculated for each participant and averaged across recorded days. Nutrient intake was evaluated in relation to dietary reference values as previously described (12, 13).

### 2.5 Laboratory analyses

Venous blood samples were collected in the morning after fasting overnight and analyzed in the laboratory within 1 h after blood sampling. An aliquot of serum was immediately stored at −80 °C for later hormonal analyses. The determination of serum biochemical and hematological parameters was performed on automatic analyzers (Chemistry Analyzer BS-240 and Hematology Analyzer BC-3600, Mindray Bio-Medical Electronics, China).

All hormonal parameters, with the exception of 25-hydroxyvitamin D and leptin (see below), were determined on an automatic analyzer using enzyme immunoassays according to the manufacturer's instructions (DRG:HYBRiD.XL®, DRG International, Inc., United States).

Serum leptin concentrations were determined using the enzyme immunoassay DRG Leptin Sandwich ELISA (EIA-2395, DRG International, Inc., United States), absorbance was detected by the Infinite F50 Plus ELISA reader (Tecan Group Ltd., Switzerland), and concentrations were calculated using software Magellan™ (Tecan Group Ltd., Switzerland).

The concentration of 25-hydroxyvitamin D was determined using a direct particle-enhanced immunoturbidimetric method on an automatic analyzer (Chemistry Analyzer BS-240, Mindray Bio-Medical Electronics, China) using diagnostic reagents according to the manufacturer's instructions (Dialab spol. s.r.o., Czech Republic).

The phase of the menstrual cycle was determined according to the following criteria: subjective evaluation of the phase of the cycle by the research participants and the concentration of luteinizing hormone (LH) helped reveal whether the woman is currently ovulating. Women with LH values greater than 15 mIU/mL (reference cut-off value for serum LH concentrations that discriminate between the follicular and luteal phase) and regular menstruation were reassigned to the ovulatory phase group. Women with LH values lower than 7 mIU/mL and regular menstruation were reassigned from the ovulatory phase group to the luteal phase (lower follicle-stimulating hormone, FSH) or the follicular phase (higher FSH) group according to the FSH value. In eumenorrheic women, blood sampling for determination of sex hormones was timed according to the phase of the menstrual cycle determined as described above, while in amenorrheic women blood sampling was performed randomly.

Two female athletic runners were supplemented with levothyroxine due to hypothyroidism. One cross-country skier, nine athletic runners, and two women in the control group were taking iron supplementation (commonly available dietary supplements in an average dose of 20 mg per day). Two cross-country skiers, nine athletic runners, and a woman in the control group were taking vitamin D supplementation (generally available dietary supplements from 1,000 IU to 2,000 IU per day). For the 25-hydroxyvitamin D, hepcidin, and iron metabolism parameters, only those who did not take vitamin D and iron supplements were compared. The study participants did not take other supplements or acute or chronic medication.

### 2.6 Statistical analyses

The Shapiro–Wilk normality test and descriptive statistics were used to evaluate the distribution of the data. Most laboratory variables did not follow normal distribution. Therefore, to ensure coherence and avoid ambiguity, all laboratory data are presented as median and interquartile range. Normally distributed data on study group characteristics (Table 1) are presented as mean ± standard deviation. Categorical data are expressed as a percentage of the specific group. The *T*-test or the Mann Whitney *U*-test was used to compare laboratory parameters. The frequency of the LEAF-Q score ≥8 or <8, absence from training/races due to injury, age of the first menstruation, frequency of menstrual cycles during the last year between athletes and a control group, and frequencies of individual genes variants between athletes and a control group were determined using Pearson's chi-square test (χ^2^). All analyses were performed with the alpha set to 0.05. Statistical analyses were performed using Statistica Ultimate Academic v. 14 (TIBCO Software Inc. California, USA).

## 3 Results

### 3.1 LEA according to LEAF-Q survey in the studied groups

Compared to the control group (*n* = 45), a higher LEAF-Q score was observed in athletes (*n* = 42) (8 vs. 5, *p* < 0.005, Table 2), with a much higher percentage of LEAF-Q score ≥8 in athletes (54.8% vs. 22.2, *p* < 0.005). Athletes also missed training sessions during the last year due to musculoskeletal problems much more common compared to the control group (57.1% vs. 33.3%, *p* < 0.05). Similarly, menstrual problems were significantly more frequently reported in athletes; 33.3% of the athletes had amenorrhea, others had oligomenorrhea, and only 31% of the athletes had a normal menstrual cycle (i.e., 12 or more cycles/year) during the year (Table 2). Athletes menstruated later than the general population; 28.6% of athletes were at the age of 15 years or older.

**Table 2:** LEAF-Q data in the initial group of athletes and control subjects.

LEAF-Q data	Female athletes *n* = 42	Control group *n* = 45	*P*-value
Age (years)	19 (17–21)	22 (20–23)	**<0.001**
LEAF-Q score	8 (4–11)	5 (2–7)	**<0.005**
LEAF-Q score <8 (*n*, %)	19 (45.2%)	35 (77.8%)	**<0.005**
LEAF-Q score ≥8 (*n*, %)	23 (54.8%)	10 (22.2%)	**<0.005**
Absence from training/races due to injury (*n*, %)	24 (57.1%)	15 (33.3%)	**<0.05**
Age of first menstruation			
I have never menstruated/ I do not remember (*n*, %)	4 (9.5%)	1 (2.2%)	NS
11 years or younger (*n*, %)	3 (7.1%)	1 (2.2%)	NS
12–14 years (*n*, %)	23 (54.8%)	40 (88.9%)	**<0.001**
15 years or older (*n*, %)	12 (28.6%)	3 (6.7%)	**<0.05**
Menstrual cycles during the last year			
I have never menstruated/My period stopped for a while now (*n*, %)	14 (33.3%)	2 (4.4%)	**<0.001**
0–2 (*n*, %)	1 (2.4%)	0	NS
3–5 (*n*, %)	4 (9.5%)	0	**<0.05**
6–8 (*n*, %)	3 (7.1%)	2 (4.4%)	NS
9–11 (*n*, %)	7 (16.7%)	20 (44.4%)	**<0.005**
12 or more (*n*, %)	13 (31%)	21 (46.7%)	NS

Data presented as a percentage of the specific group in the column: *n* (%) or as median and interquartile range. Bold values indicate statistically significant results.

LEAF-Q, the low energy availability in females questionnaire.

As described above, according to the questionnaire data, the subjects were divided into a group of female athletes with a LEAF-Q score ≥8 (*n* = 23) and a group of control women who had a LEAF-Q score <8 (*n* = 21).

### 3.2 Characteristics of the groups according to LEAF-Q score

The anthropometric characteristics of the studied groups are given in Table 1. Compared to the control group with LEAF-Q < 8, athletes with the LEAF-Q score ≥8 had lower body fat (19.9 vs. 22.4%, *p* < 0.005), BMI (19.7 vs. 22.2 kg/m^2^, *p* < 0.001) and weight (56.8 vs. 61.5 kg, *p* < 0.05). The athletes were taller (170 vs. 166 cm, *p* < 0.05) and spent more time training (12 vs. 5 h/week, *p* < 0.001).

### 3.3 Nutrition and energy expenditure

Compared to control women with LEAF-Q < 8, total and exercise energy expenditure was higher (*p* < 0.001) in athletes with LEAF-Q ≥ 8 and athletes had a negative energy balance (90%) with a low EA value (24,3 kcal/kg FFM/day) (Table 3). Surprisingly, the control women also, although with a low LEAF-Q score and steady energy balance, had a borderline value of EA (29.8 kcal/kg FFM/day). Interestingly, the dietary intake of vitamin D, iron, and folate was below the recommended daily intake (RDI) in both groups (Table 3). However, in a group of athletes, there was a higher dietary intake of iron (*p* < 0.05) compared to the control group and the athletes paid more attention to iron and vitamin D supplementation than a control group (10 athletes vs. 2 women in the control group took iron supplementation, 11 athletes vs. 1 woman in the control group took vitamin D supplementation). Compared to controls, athletes had a higher consumption of carbohydrates (*p* < 0.05), and proteins (*p* < 0.001) and a lower intake of fats (*p* < 0.001) (Table 3). However, the athletes still had an inadequate recommended dietary allowance (RDA) for carbohydrate intake (Table 3).

**Table 3:** Nutrition analysis in athletes and control subjects.

Nutrition information		Female athletes LEAF-Q score ≥8 *n* = 20	Control group LEAF-Q score <8 *n* = 21	*P*-value
Energy	Energy intake (kJ/day)	9.272 (7.49–10.48)	8.247 (7.30–8.59)	NS
	Energy intake (kcal/day)	2.216 (1.79–2,51)	1.971 (1.74–2,05)	NS
	Energy balance (%)	89.7 (74.8–111)	100 (94.4–109)	NS
	Total energy expenditure (kcal/day)	2.399 (2.24–2.49)	1.884 (1.860–1.952)	**<0.001**
	Exercise energy expenditure (kcal/day)	1,008 (942–1,045)	514 (507–532)	**<0.001**
	Energy availability (kcal/kg FFM/day)	24.3 (16.9–35.8)	29.8 (26.5–33.3)	NS
Proportion of macronutrients to total caloric intake and weight	Carbohydrates (%) (RDA: 55%–65%)	51 (47–54)	47 (43–49)	**<0.05**
	Carbohydrates (g/kg) (RDA: 5–8 g/kg/day)	4.8 (3.8–6.5)	3.6 (3.3–3.8)	**<0.005**
	Proteins (%) (RDA: 15%–20%)	18 (17–23)	17 (16–19)	NS
	Proteins (g/kg) (RDA: 1.2–1.7 g/kg/day)	1.7 (1.6–2.3)	1.3 (1.2–1.5)	**<0.001**
	Fats (%) (RDA: 20%–30% of total caloric intake)	30 (27.5–32.5)	36 (34–38)	**<0.001**
Dietary intake of micronutrients	Vitamin D (IU) (RDI: 200–600 IU)	82.8 (53.3–214)	75.5 (58.5–105)	NS
	Iron (mg) (RDI: 18 mg)	12.6 (10.8–14.7)	9.3 (7.6–12.7)	**<0.05**
	Folates (µg) (RDI: 400 µg)	237 (197–314)	212 (140–226)	NS

Data presented as median and interquartile range. Bold values indicate statistically significant results.

Energy balance, energy intake/total energy expenditure × 100; RDI, recommended daily intake for female athletes; RDA, recommended dietary allowance for athletes.

### 3.4 Blood serum parameters

The blood serum biomarkers are given in Table 4. As described above for the 25 hydroxyvitamin D, hepcidin, and iron metabolism parameters, only those who did not supplement were compared. Concentrations of 25 hydroxyvitamin D were at the lower end of the reference range and comparable between both groups. Compared to the control group with LEAF-Q score <8, athletes with LEAF-Q score ≥8 had lower serum concentrations of leptin (*p* < 0.001), white blood cells (*p* < 0.005) and phosphorus (*p* < 0.005), and higher concentrations of hepcidin (*p* < 0.05) and free T3 (*p* < 0.05). The concentrations of free T4 and TSH did not differ and were within the reference limits. Hypophosphatemia (serum phosphorus concentration ≤0.8 mmol/L) occurred only in 3 athletes. The fasting serum leptin concentration was lower in the athlete group (*p* < 0.001) and was positively correlated with adiposity (percentage of body fat) (*r* = 0.65, *p* < 0.001) and negatively correlated with time spent on training/exercising (*r* = −0.5, *p* < 0.05) in all subjects.

**Table 4:** Blood parameters in athletes and control subjects.

Panel of blood parameters	Marker (reference range)	Female athletes LEAF-Q score ≥8 *n* = 23	Control group LEAF-Q score <8 *n* = 21	*P*-value
Hormones	25-hydroxyvitamin D (50–180 nmol/L)	60.9 (52–85.6)	52.7 (48.4–67.3)	NS
	Estradiol (pmol/L)	171 (84–225)	274 (225–432)	**<0.001**
	Progesterone (nmol/L)	1.42 (1.24–1.93)	4.53 (2.26–10.5)	**<0.001**
	FSH (mIU/mL)	5.33 (3.08–7.39)	4.55 (2.92–7.08)	NS
	LH (mIU/mL)	6.59 (3.06–13.1)	6.6 (4.27–12.5)	NS
	Free testosteron (1–9.9 pmol/L)	6.01 (4.42–7.87)	6.47 (4.8–7.43)	NS
	Cortisol (138–635 nmol/L)	507 (458–584)	547 (504–636)	NS
	TSH (0.35–4.94 µIU/mL)	2.05 (1.66–2.79)	2.38 (1.79–2.88)	NS
	Free T3 (2.58–5.44 pmol/L)	3.54 (2.74–4.44)	2.68 (2.02–3.31)	**<0.05**
	Free T4 (9–19 pmol/L)	13.8 (9.37–15.4)	11.3 (11–12.3)	NS
	Insulin (3.56–22.1 µIU/mL)	9.62 (7.74–15.9)	11.9 (7.54–15.2)	NS
	Hepcidin (0.2–30.7 ng/mL)	5.15 (2.67–5.96)	0.96 (0.19–2.9)	**<0.05**
	Leptin (0.7–8.3 ng/ml)	2.01 (1.11–3.51)	7.05 (3.37–8.25)	**<0.001**
Lipid panel	Total cholesterol (2.9–5.2 mmol/L)	4.47 (3.83–5.56)	4.34 (3.72–4.51)	NS
	HDL cholesterol (1–2.1 mmol/L)	1.7 (1.53–2.14)	1.64 (1.48–1.75)	NS
	LDL cholesterol (1.2–3 mmol/L)	2.37 (1.78–2.85)	1.9 (1.64–2.44)	NS
	Triacylglyceroles (<2.3 mmol/L)	0.62 (0.48–0.73)	0.61 (0.54–0.8)	NS
Complete blood count	White blood cells (4–10 × 10^9^/L)	5 (4.5–5.7)	5.9 (5.3–6.9)	**<0.005**
	Red blood cells (3.5–5.5 × 10^12^/L)	4.49 (4.28–4.73)	4.48 (4.32–4.72)	NS
	Hemoglobin (120–160 g/L)	137 (132–143)	135 (129–143)	NS
	Hematocrit (0.37–0.46)	0.41 (0.39–0.42)	0.41 (0.38–0.43)	NS
Iron metabolism	Iron (6.6–28 µmol/L)	16 (11.9–19.3)	12.8 (6.29–17.6)	NS
	Ferritin (8–150 µg/L)	28 (17–49)	19.5 (10.2–34.5)	NS
	Transferrin (2–3.6 g/L)	2.9 (2.5–3.4)	2.64 (2.51–3.21)	NS
	Iron saturation of transferrin (16–45%)	21.2 (19.2–28)	20.4 (9.1–26.6)	NS
Metabolic panel	Glucose (3.9–5.6 mmol/L)	4.85 (4.58–5.01)	4.54 (4.48–4.81)	NS
	Total protein (66–88 g/L)	72.7 (68.2–76.5)	71.4 (68.9–73)	NS
	Albumin (35–53 g/L)	46 (44–47.4)	45.9 (43.6–47.8)	NS
	Prealbumin (0.2–0.4 g/L)	0.26 (0.21–0.3)	0.25 (0.23–0.27)	NS
	Urea (2–6.7 mmol/L)	5.68 (4.4–7.43)	4.72 (4.42–5.4)	NS
	Creatinine (44–104 µmol/L)	72.3 (66.5–80.2)	72.6 (66.9–82.2)	NS
	Bilirubin (2–21 µmol/L)	9.5 (7.2–12.8)	10.1 (9.06–15.8)	NS
Ions	Calcium (2.15–2.65 mmol/L)	2.39 (2.3–2.48)	2.35 (2.33–2.45)	NS
	Phosphorus (0.81–1.45 mmol/L)	1.02 (0.9–1.17)	1.11 (1.07–1.26)	**<0.005**

Data presented as median and interquartile range. For athletes *n* = 23, except for determination of estradiol (*n* = 22), insulin (*n* = 21), and leptin (*n* = 21). For 25-hydroxyvitamin D, hepcidin and parameters of iron metabolism, only those who did not supplement were compared (25-hydroxyvitamin D, *n* = 12 for athletes and *n* = 20 for the control group; hepcidin and parameters of iron metabolism, *n* = 13 for athletes and *n* = 19 for the control group). Bold values indicate statistically significant results.

The reference range for estradiol, progesterone, FSH a LH depends on the phase of the cycle.

FSH, follicle-stimulating hormone; LH, luteinizing hormone; TSH, thyroid-stimulating hormone; T3, triiodothyronine; T4, thyroxine.

Athletes also had lower estradiol and progesterone concentrations compared to the control group (*p* < 0.001) (Table 4). Almost 50% of the athletes had an irregular or absent menstrual cycle. In accord, the estradiol concentration in athletes generally moved around the lower reference range in individual phases of the cycle (Table 5). There were lower concentrations of progesterone (*p* < 0.05) in the luteal phase in athletes compared to the control group, and this concentration was below the lower limit of the reference range (Table 5). When compared eumenorrheic and amenorrhoeic/oligomenorrheic subjects, significantly different concentrations of estradiol (239 pmol/L vs. 117 pmol/L, *p* < 0.005) and progesterone (2.68 nmol/L vs. 1.41 nmol/L, *p* < 0.005) were observed (Table 6).

**Table 5:** Concentration of estradiol and progesterone according to the menstrual cycle in athletes and control subjects.

Hormones/cycle phase	Female athletes LEAF-Q score ≥8 *n* = 23	Control group LEAF-Q score <8 *n* = 21	*P*-value
Estradiol (reference range)	Concentration		
Follicular phase (45–854 pmol/L)	190 (107–228) *n* = 6	240 (214–331) *n* = 7	NS
Ovulation (151–1,461 pmol/L)	218 (184–610) *n* = 4	594 (239–933) *n* = 5	NS
Luteal phase (82–1,251 pmol/L)	124 (78–170) *n* = 2	273 (225–405) *n* = 9	NS
Irregular/absence of menstrual cycle	117 (84–184) *n* = 10	–	
Progesterone (reference range)	Concentration		
Follicular phase (0.2–2.8 nmol/L)	1.7 (1.24–2.38) *n* = 6	2.19 (1.44–2.79) *n* = 7	NS
Ovulation (0.4–38 nmol/L)	2.33 (1.52–43.98) *n* = 4	3.62 (2.68–10.5) *n* = 5	NS
Luteal phase (5–76 nmol/L)	1.23 (1.17–1.29) *n* = 2	5.6 (4.54–11.78) *n* = 9	**<0.05**
Irregular/absence of menstrual cycle	1.41 (1.26–1.65) *n* = 11	–	

Data presented as median and interquartile range. Bold values indicate statistically significant results.

RR, reference ranges.

**Table 6:** Concentration of hormones according to problem with menstrual cycle.

Hormones	Eumenorrheic female (*n* = 33)	Amenorrheic/oligomenorrheic female (*n* = 11)	*P*-value
Estradiol (pmol/L)	239 (185–405)	117 (84–184)	**<0.005**
Progesterone (nmol/L)	2.68 (1.83–5.61)	1.41 (1.26–1.65)	**<0.005**
Leptin (ng/mL)	4.24 (2.66–7.34)	1.93 (0.74–3.65)	***p*** **=** **0.05**
FSH (mIU/mL)	4.69 (3.08–7.08)	5.33 (2.86–7.45)	NS
LH (mIU/mL)	7.14 (4.26–12.8)	4.54 (2.27–9.25)	NS
Testosteron free (pmol/L)	6.47 (4.81–7.43)	4.54 (3.52–8.1)	NS
Cortisol (nmol/L)	530 (458–591)	544 (478–707)	NS
TSH (µIU/ml)	2.38 (1.56–2.88)	1.93 (1.79–2.79)	NS
T3 free (pmol/L)	3.21 (2.46–3.9)	3.68 (2.24–5.97)	NS
T4 free (pmol/L)	11.4 (10.9–12.9)	14.3 (9.06–16)	NS
Insulin (µIU/mL)	11.9 (7.93–15.9)	9.4 (4.67–12.1)	NS
Hepcidin (ng/mL)	1.61 (0.58–5.08)	4.54 (1.5–5.96)	NS
25-hydroxyvitamin D (nmol/L)	60.2 (50–70.8)	63.1 (54.1–80.7)	NS

Data presented as median and interquartile range. For amenorrheic/oligomenorrheic subject *n* = 11, except for determination of estradiol (*n* = 10), insulin (*n* = 9), and leptin (*n* = 9).

Bold values indicate statistically significant results.

FSH, follicle-stimulating hormone; LH, luteinizing hormone; TSH, thyroid-stimulating hormone; T3, triiodothyronine; T4, thyroxine.

## 4 Discussion

The objective of the current study was to evaluate markers associated with RED-S in a cohort of female athletes. Menstrual dysfunction is common in female athletes but is often ignored and considered a natural result of intense training, despite the risk of negative health consequences (14). In our study, only 31% of the athletes in the initial cohort had normal menstrual cycles (i.e., 12 or more cycles/year) during the year and 33.3% of the athletes had amenorrhea, others had oligomenorrhea (Table 2). Several studies indicate that knowledge of the female hormonal cycle is insufficient between athletes and coaches, and communication is also often difficult. In a study by Solli et al., only 8% of 140 women athletes (cross-country skiing/biathlon) stated that they had sufficient knowledge of the menstrual cycle in relation to training and performance (15), despite the fact that nutritional interventions can restore the normal cycle in some female athletes (16).

Exercise *per se* does not have a suppressive effect on reproductive functions. However, athletes with functional hypothalamic amenorrhea generally have lower EA than eumenorrheic athletes, and menstrual disturbances increase linearly as EA decreases (17). Pulsatility of LH has also been reported to be altered when an EA threshold has been <30 kcal/kg FFM/day (18). Furthermore, there is also a decrease in the drive of gonadotropin-releasing hormones with a reduction in the frequency of FSH and LH pulsatility, leading to changes in folliculogenesis and ovulatory function, resulting in lower concentrations of estradiol and progesterone (19), findings also observed in our study (Table 4).

Maintaining EA throughout the day is also critical for endurance athletes to maintain performance and reduce the risk of overtraining and associated problems. Although a significant difference in energy expenditure was observed in our athletes, a lower EA alone does not appear to be a reliable marker for evaluating LEA. This is in agreement with a study by Burke et al., who raised doubts about the reliability and validity of existing methods to measure EA in athletes, suggesting that laboratory markers can be beneficial in monitoring and detecting the early phases of EA (3), and their implementation in standardized methodologies should improve the assessment of EA (1). Additionally, in our study, athletes with a high LEAF-Q score had insufficient carbohydrate intake, which can further limit their performance and slow down regeneration processes.

Athletes generally do not meet the daily adequate intake of vitamin D (20). The importance of adequate intake of vitamin D is widely recognized, particularly for musculoskeletal and immune systems (20). Another important micronutrient is iron. Iron deficiency can compromise reproductive functions, bone health, and negatively affect thyroid functions (21). Iron stores affect aerobic performance, as reported in a study by McClung et al. in female soldiers (22). In our study, the dietary intake of vitamin D, iron and folate was below the RDI in both groups (Table 3) although the athletes paid more attention to iron and vitamin D supplementation than the control subjects.

Despite slightly higher free T3 concentrations observed in our athletes, no differences were found between eumenorrheic and amenorrheic/oligomenorrheic women (Table 6). Strenuous exercise can be associated with transient alterations in thyroid hormones (23). Long-lasting amenorrhea is known to significantly reduce serum free T3 concentrations in athletes (24); thyroid dysfunction can also be induced by nutritional factors, including insufficient energy intake and iodine, selenium, iron, and vitamin D deficiency (23), but this was not observed in our study.

Exercise-induced inflammation negatively affects iron absorption in the gastrointestinal tract by up-regulating hepcidin, the primary regulator of iron absorption. Interestingly, hepcidin appears to be a potential biomarker of LEA (25). In fact, an increase in hepcidin concentrations was positively associated with energy expenditure and negatively associated with energy balance in post-military training (26). In our study, athletes with LEAF-Q ≥ 8 had significantly higher concentrations of hepcidin (Table 4) compared to a control group with LEAF-Q score <8.

Our athletes also had lower serum leptin concentrations, which was positively correlated with adiposity and negatively correlated with time spent training. There were no significant differences between eumenorrheic and amenorrheic/oligomenorrheic subjects (Table 6). Physiologically, leptin responds to a negative energy balance and decreased energy stores (27). Conditions in which nutritional status is suboptimal, such as eating disorders, exercise-induced amenorrhea, and functional hypothalamic amenorrhea, are associated with low serum leptin concentrations that indicate a direct link between adipose tissue and the reproductive system (27).

Athletes with LEAF-Q score ≥8 had lower serum phosphorus concentrations than the control group with a LEAF-Q score <8, (Table 4); apparent hypophosphatemia (phosphorus ≤0.8 mmol/L) occurred in 3 athletes (13%). Phosphate plays a critical role in skeletal mineralization, energy homeostasis, enzyme function, cell membrane integrity, and neurologic function and is under the control of vitamin D. Interestingly, hypophosphatemia has been used as a marker of malnutrition (28).

Furthermore, white blood cell count was lower in our athletes (Table 4). However, the reduction in white blood cells does not appear to be related to an energy deficit, but rather to a sports load. Intense exercise can cause immune deficiency and increased susceptibility to infectious complications (29).

Our study has several limitations. The cohort size of the athletes and the control population was small and did not enable us to perform adjustment for possible confounding factors. As the study was not designed based on an *a priori* sample size calculation, its statistical power is limited, particularly for detecting small effects. The results should therefore be interpreted as exploratory, with particular caution when considering non-significant findings. The control population differed in age and had healthy lifestyle habits, including regular exercise and healthy food intake, indicating that the control women did not represent the general population in which the sedentary lifestyle is predominant. Other limitations include the fact that the dietary intake was calculated from self-reported records, which can underestimate the energy and nutrient intake, especially in athletes at risk of LEA. Furthermore, the total and exercise energy expenditure in the present study was estimated using predictive equations and fixed physical activity factors. Although this approach is commonly used in field-based measurements, this method can increase the risk of misclassification of energy availability due to individual variability in training load and non-exercise activity. Our study was also not designed to assess direct bone-related outcomes, which is another limitation of our study. Finally, the athletes were in different phases of the annual training cycle when enrolled in the study.

In conclusion, LEA is prevalent in female endurance athletes and its diagnosis deserves a multifactorial approach with anthropometric and nutritional analyses, and a wider range of laboratory markers to be used, including the assessment of menstrual dysfunction (questionnaire/hormone concentration). A proper nutritional plan is essential for athletes to achieve an optimal intake of micro- and macronutrients and a proper energy balance.

## Data Availability

Datasets are available on request, the raw data supporting the conclusions of this article will be made available by the authors, without undue reservation.
